# The reciprocal relationship between episodic memory and future thinking: How the outcome of predictions is subsequently remembered

**DOI:** 10.1002/brb3.2603

**Published:** 2022-08-24

**Authors:** Sneh P. Patel, Matthew P. McCurdy, Andrea N. Frankenstein, Allison M. Sklenar, Pauline Urban Levy, Karl K. Szpunar, Eric D. Leshikar

**Affiliations:** ^1^ University of Illinois at Chicago Chicago Illinois USA; ^2^ Ryerson University Toronto Ontario Canada

**Keywords:** episodic memory, future thinking, recall, recognition, social cognition

## Abstract

Evidence suggests that memory is involved in making simulations and predictions about the future (i.e., future thinking), but less work has examined how the outcome of those predictions (whether events play out as predicted or expected) subsequently affects episodic memory. In this investigation, we examine whether memory is better for outcomes that are consistent with predictions, or whether memory is enhanced for outcomes that are inconsistent with predictions, after the predicted event occurs. In this experiment, participants learned a core trait associated with social targets (e.g., high in extroversion), before making predictions about behaviors targets would perform. Participants then were shown behaviors the social targets actually performed (i.e., prediction outcome), which was either consistent or inconsistent with predictions. After that, participants completed a memory test (recognition; recall) for the prediction outcomes. For recognition, the results revealed better memory for outcomes that were consistent with traits associated with targets (i.e., trait‐consistent outcomes), compared to outcomes that were inconsistent (i.e., trait‐inconsistent outcomes). Finding a memory advantage for trait‐consistent outcomes suggests that outcomes that are in line with the contents of memory (e.g., what one knows; schemas) are more readily remembered than those that are inconsistent with memory, which may reflect an adaptive memory process. For recall, memory did not differ between trait‐consistent and trait‐inconsistent outcomes. Altogether, the results of this experiment advance understanding of the reciprocal relationship between episodic memory and future thinking and show that outcome of predictions has an influence on subsequent episodic memory, at least as measured by recognition.

## Graphical abstract

In this investigation, we examined the relationship between future thinking and episodic memory. Specifically, we investigated whether the outcome of predictions, whether events occurred as predicted, has an effect on memory after the predicted events play out. The results showed substantially better memory for events that occurred as predicted, compared to events that did not occur as predicted, which advances the understanding of the relationship between future thinking and memory.

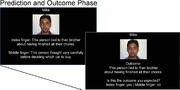

## 1 INTRODUCTION

In recent years, there has been growing scientific interest in future thinking (Schacter et al., 2017). Future thinking is an important construct involved in planning, decision‐making, problem‐solving, and other tasks (Schacter, 2019; Schacter et al., 2017). Work on future thinking suggests that people use memory processes related to retrieving past experiences (episodic memory) to simulate or predict what might happen in the future (D'Argembeau, 2012; Schacter & Addis, 2007; Schacter et al., 2008). This relationship between episodic memory and episodic future thinking has been demonstrated in work in amnesics (Hassabis et al., 2007), neuroimaging evidence showing overlapping cortical activity (Addis et al., 2007; Schacter et al., 2007; Szpunar et al., 2007), and through behavioral evidence (Schacter et al., 2017). For instance, past behavioral work has shown that episodic future thinking is more perceptually vivid when simulations occur in contexts more recently experienced, compared to contexts experienced in the more remote past (Szpunar & McDermott, 2008), similar to how episodic memory is typically better for more recent, compared to more remote, experiences. Although this past work has been essential in understanding how episodic memory is involved in different types of episodic future thinking, such as making predictions, less work has examined how the outcome of predictions (e.g., whether events play out as predicted) affects episodic memory once an event occurs. In this investigation, we examined memory for behaviors (e.g., outcomes) that are either consistent (henceforth called *trait‐consistent outcome*) or inconsistent (*trait‐inconsistent outcome*) with predictions based on core traits associated with targets to better understand how the outcome of predictions is subsequently remembered. We do so in a task that involves participants making predictions about social targets, which is in line with past work showing that people frequently and spontaneously engage in making future predictions about social targets (Tamir & Thornton, 2018).

Given our focus on memory for trait‐consistent versus trait‐inconsistent prediction outcomes involving social targets, it is first informative to look at past work on memory effects for consistent versus inconsistent information about social targets in tasks that do not involve making predictions. In the social literature, there has been extensive work showing that information that is inconsistent with what is already known about social targets is more memorable than consistent information (Hamilton & Sherman, 1996; Hamilton et al., 2003; Hastie & Kumar, 1979; Rojahn & Pettigrew, 1992; Skowronski et al., 2013; Srull, 1981; Stangor & McMillan, 1992). In a seminal investigation, participants were asked to learn about social targets that had a specific personality trait and were then given sentences that described behaviors targets performed. These behaviors were consistent, inconsistent, or neutral with respect to the personality trait of the target. The results showed that inconsistent behaviors were better remembered than consistent or neutral behaviors (Hastie & Kumar, 1979). In another experiment, participants were first given either positive (honest, friendly) or negative (dishonest, unfriendly) information about targets and then were shown additional behaviors that were either consistent or inconsistent with that initial information. The results showed that inconsistent behaviors associated with targets were better remembered than consistent behaviors (Ybarra & Stephan, 1996). Such a finding of enhanced memory for inconsistent relative to consistent information about targets is further in line with several comprehensive meta‐analyses that have found such effects across a wide range of studies (Rojahn & Pettigrew, 1992; Srull, 1981; Stangor & McMillan, 1992). Although this past work has not focused on making predictions about social targets, these findings and others demonstrate that inconsistent information about social targets is generally well‐remembered. Interestingly, work in another domain that does focus on making predictions about social targets (so‐called “predictive processing models”; Bach & Schenke, 2017; Otten et al., 2017) further supports the idea that information inconsistent with what one predicts/expects can lead to updating of information about those targets (in line with the idea that inconsistent information may be well‐remembered). Specifically, theoretical work on predictive processing models suggests that people spontaneously make predictions of future actions targets may engage in (based on prior knowledge) but that when those predictions are violated, this results in a prediction error that can then lead to updating knowledge about the social target. Most relevant to the current study, this past work may suggest that outcomes that are inconsistent with predictions based on traits (e.g., trait‐inconsistent outcomes) will be better remembered than outcomes consistent with predictions based on traits. Such a result (e.g., prediction inconsistent memory advantage) would further be aligned with evidence suggesting that outcomes that are not expected or surprising tend to be well remembered (Antony et al., 2021; Greve et al., 2017; Rouhani et al., 2021, 2018). Such an advantage for inconsistent outcomes may be adaptive because remembering information that is inconsistent with the contents of memory stores (about targets) might allow for memory to be adjusted in a way that may make future predictions more accurate.

Although abundant work has shown better memory for information inconsistent with the contents of memory in the social domain, there has been other work (in tasks that do not involve making predictions) that argues consistent information associated with social targets is better remembered than inconsistent information (Cohen, 1981; Frey & Smith, 1993; Rothbart et al., 1979; Synder & Uranowitz, 1978). This past work showing improved memory for consistent versus inconsistent information has often been interpreted through the concept of schemas or schematic representations of targets (Judd & Kulik, 1980; Rothbart et al., 1979; Synder & Uranowitz, 1978). Past theoretical work suggests that people develop schemas based on prior experiences involving targets (e.g., that a person is extroverted) and use that schematic representation to process new information about those same targets (Taylor & Crocker, 1981; Wyer & Martin, 1986; Wyer & Srull, 1989). Specifically, schema accounts suggest that new information consistent with existing schematic representations (e.g., memory) of targets is more easily integrated into existing memory stores, compared to information that does not fit existing schemas (i.e., inconsistent information), yielding improved memory for consistent information. As one example of this idea, participants in one investigation were shown targets that performed behaviors that were either friendly *or* unfriendly. Participants were then shown additional behaviors performed by targets, but this time, targets performed both friendly and unfriendly behaviors. The results showed that participants remembered the consistent behaviors performed by targets better than inconsistent behaviors (Frey & Smith, 1993). In another investigation, participants watched a video of targets exhibiting prototypical characteristics of a librarian and a restaurant server. Half the participants were told that the social target was a librarian, and the other half were told that the social target was a server. The results revealed better memory for prototypically consistent relative to prototypically inconsistent behaviors performed by targets (Cohen, 1981), aligned with the idea that schema‐consistent information shows an advantage in memory. Although this past work did not involve participants making predictions about social targets, this work leads to the hypothesis that trait‐consistent outcomes (i.e., behaviors consistent with earlier predictions based on core traits of targets) may be better remembered than trait‐inconsistent outcomes since such information may be more easily integrated into existing memory stores. Given that schematic representations are generally adequate representations of the world, it may be adaptive to rely on existing schemas when processing new information about targets.

Up to this point, we have described evidence that would support the hypothesis that memory would be better for *trait‐inconsistent outcomes* as well as a different body of evidence that might suggest better memory for *trait‐consistent outcomes*. In one of the only investigations to directly examine the effects of prediction outcome on subsequent episodic memory (Frankenstein et al., 2020), participants learned a core trait (e.g., extroversion) about various social targets before making predictions about which of two behaviors targets were most likely to perform: one behavior was consistent with the core trait associated with targets, whereas the other behavior was inconsistent with the core trait. After making a prediction about which behavior the target would perform, participants were told the behavior the target actually performed (i.e., prediction outcome). Social targets performed both trait‐consistent outcomes (e.g., behaviors consistent with the core trait of targets) and trait‐inconsistent outcomes (e.g., behaviors inconsistent with the core trait of targets). Finally, participants completed a recognition memory test where they reported which behavior the target actually performed (i.e., prediction outcome). The results showed a consistency advantage in subsequent memory, where memory was better for trait‐consistent outcomes than for trait‐inconsistent outcomes, suggesting that participants were better able to integrate outcomes that fit with the existing contents of memory. These results are in line with past work demonstrating that information that is congruent with existing schemas about targets is more memorable than inconsistent information (Cohen, 1981; Frey & Smith, 1993; Hamilton & Garcia‐Marques, 2003; Rothbart et al., 1979; Synder & Uranowitz, 1978; Taylor & Crocker, 1981; Wyer & Srull, 1989). In this investigation, we extend the work of Frankenstein et al. (2020) by examining memory for prediction outcomes as measured by recognition as well as by recall measures, and we do so in a task where social targets were associated with a wider range of core traits to understand the extent that memory might be enhanced for trait‐consistent or trait‐inconsistent outcomes for a variety of different types of behavioral information (i.e., different trait information). Thus, the results of the current investigation will replicate and extend our past work (Frankenstein et al., 2020) and contribute to a better understanding of the relationship between future thinking and episodic memory.

In this investigation, we examine memory for the outcome of predictions that are either consistent (i.e., trait‐consistent outcomes) or inconsistent (trait‐inconsistent outcomes) with what one predicts to better understand the relationship between predictions (e.g., future thinking) and episodic memory. To do so, participants were asked to learn a single core trait about social targets (e.g., highly agreeable). Participants were then asked to make predictions about behaviors targets would perform before being shown the actual behavior targets performed (i.e., prediction outcome), which was either consistent or inconsistent with the core trait of that target. Participants then completed a memory test (both recall and recognition) for the actual behaviors targets performed (i.e., prediction outcome). We expected one of two possible results in this investigation. First, given that abundant past work suggests that information inconsistent with what is known about targets is more memorable than consistent information (Hastie & Kumar, 1979; Rojahn & Pettigrew, 1992; Srull, 1981; Stangor & McMillan, 1992), we thought it possible that trait‐inconsistent outcomes would be better remembered than trait‐consistent outcomes. Such a finding would further be consistent with other work suggesting that unexpected or surprising information exhibits enhanced memory (Antony et al., 2021; Greve et al., 2017; Rouhani et al., 2021, 2018). Second, an alternative possibility is that trait‐consistent outcomes would be better remembered than trait‐inconsistent outcomes. This possibility would be aligned with theoretical work on the influence of schematic representations on memory (in tasks that do not involve predictions), which suggests that information that is consistent with what is already known is easier to integrate into memory and thus more memorable (Taylor & Crocker, 1981; Wyer & Martin, 1986). Finding support for either hypothesis would advance knowledge about future thinking (e.g., predictions) by showing how the outcome of predictions is subsequently stored in episodic memory after the predicted event occurs.

## 2 METHOD

### 2.1 Participants

Twenty‐three young adults (11 females, *M*_age_: 18.5, *SD*_age_: 0.08) were recruited to participate from the University of Illinois at Chicago. A power analysis based on results from piloting showed that a sample of 17 participants would be sufficient to achieve a power of 0.80 at an alpha of .05.^1^ Informed written consent was provided by all participants in adherence with the university's Institutional Review Board. Participants were given course credit for participating in this experiment.

### 2.2 Stimuli

Sixteen faces (Minear & Park, 2004) and 16 names (half male, half female) were used as stimuli. In addition, we used 160 normed behavioral sentences (Fuhrman et al., 1989). The sentences depicted behaviors that expressed either high or low amounts of four of the Big Five traits (high and low dimensionality for each: agreeableness, conscientiousness, extroversion, and openness to experience). In our past work (Frankenstein et al., 2020), we used sentences that implied extroversion and openness to experience, and in the current stimuli, we added behaviors implying conscientiousness and agreeableness to better understand memory effects for prediction outcomes for other types of trait information associated with targets. Furthermore, sentences were constructed to have two or three idea units, following past work (Limbert et al., 2018), which would allow participants the opportunity to remember multiple details associated with each behavior performed by targets. For example, a sentence that reflects high extroversion, such as “This person went over to greet the new neighbor that had moved in next door,” was composed of three idea units: “went over to greet,” “new neighbor,” and “moved in next door.” Behavioral sentences were counterbalanced to appear with different social targets across participants.

### 2.3 Procedure

Participants completed the experiment in a single laboratory session. The experiment consisted of three phases: person learning, prediction and outcome, and memory test, similar to our prior work (Frankenstein et al., 2020). In the person learning phase, participants were asked to study behavioral statements of about 16 social targets. Participants were shown six behavioral statements that all implied a single core trait (e.g., high agreeableness, low agreeableness, high conscientiousness, low conscientiousness, etc.) for each of the 16 targets. All six of the behavioral sentences were shown at the same time, along with the face and name of the target (see the Appendix). To ensure that participants were learning behaviors associated with each target, they studied the behaviors for each social target twice. The person learning phase was self‐paced. During person learning, participants were unaware of the prediction and outcome phase or the memory test phase.

**FIGURE 1:**
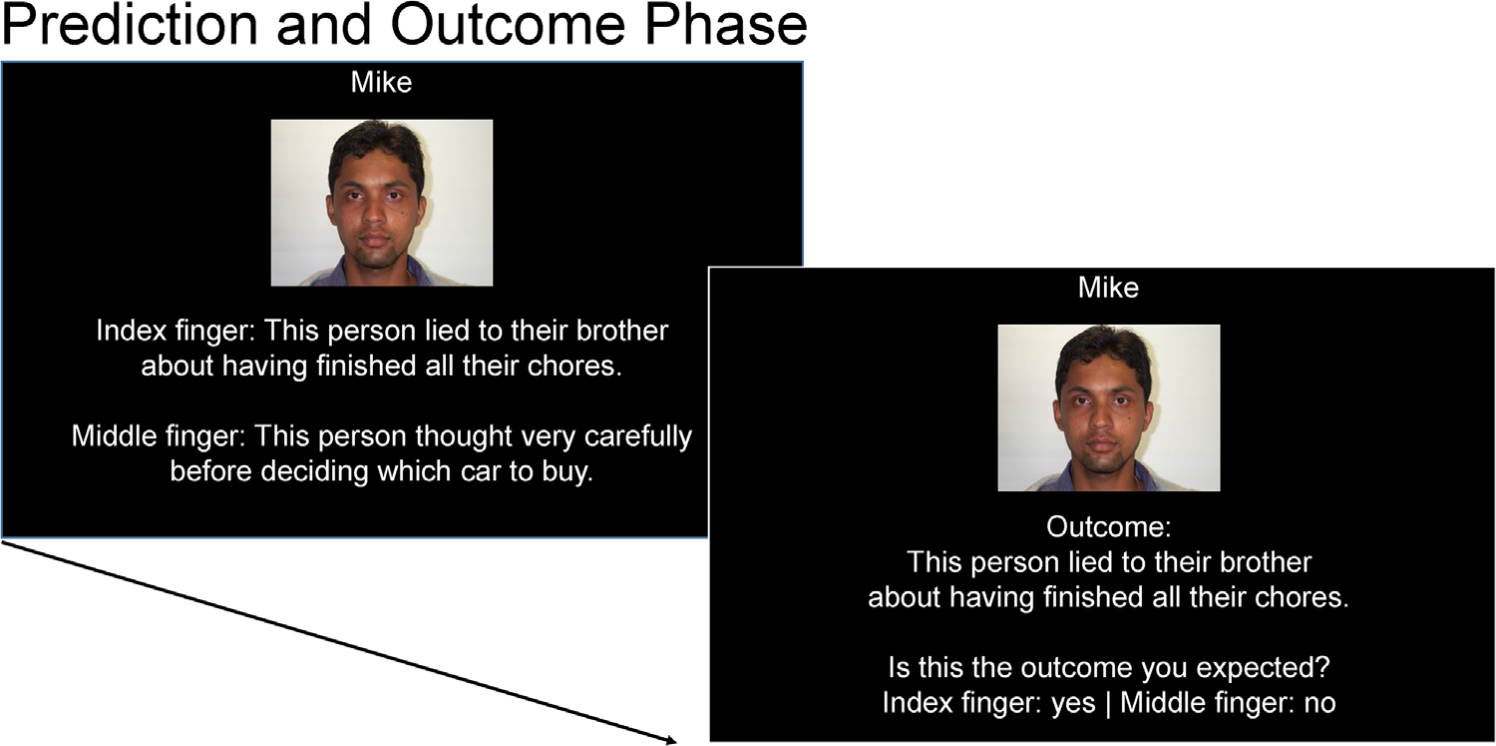
Depiction of a trial from the prediction and outcome phase of the experiment

Next, participants completed the prediction and outcome phase. In each prediction and outcome phase trial, participants were shown the name and face of one of the targets they studied in the person learning phase along with two behavioral sentences (see Figure 1). One of the behaviors was consistent with the core trait associated with that target (in the person learning phase), and the other statement was inconsistent. Participants were asked to predict which of the two behaviors the target was most likely to perform. Immediately after they selected the behavior (i.e., made their prediction), participants were shown the behavior the target actually performed (i.e., *prediction outcome*) and were asked to decide whether they expected that behavior (yes or no). Participants completed 32 trials in this phase of the experiment (making two predictions for each social target). Each trial contained two unique behaviors (i.e., two behaviors not seen before). In half of the trials, the prediction outcome was consistent with the core trait for each target (i.e., *trait‐consistent outcome*), and in the other half, the prediction outcome was inconsistent (i.e., *trait‐inconsistent outcome*).^2^ Thus, each of the 16 social targets was associated with both a trait‐consistent and a trait‐inconsistent outcome. Because each social target was associated with both a trait‐consistent and a trait‐inconsistent outcome, we counterbalanced the order of outcomes such that for half the targets (i.e., eight of the targets), a trait‐consistent outcome was shown first, whereas for the other half of targets, a trait‐inconsistent outcome was shown first. We did this to balance out any possible effects on memory of seeing a trait‐consistent versus a trait‐inconsistent outcome first.

After the prediction and outcome phase, participants completed a memory test phase that consisted of a recognition memory test, followed by a recall test. Starting first with the recognition test, participants were asked to complete 32 recognition trials where they identified the behavior that was actually performed (i.e., prediction outcome) by social targets. For each recognition trial, participants saw a target as well as the same two behaviors from the prediction and outcome phase of the experiment and were asked to report which of the two behaviors the social target actually performed. Instructions made it clear that participants were not making a prediction again, but instead they were asked to report which behavior the social target actually performed. Recognition was self‐paced. Following recognition, participants then completed a recall test. In the recall test, participants were given a separate sheet of paper for each of the 16 targets, with the face and name of a single target shown on each page. Under the face name, there were two sets of lines where participants were asked to write down the two behaviors the individual actually performed. Specifically, participants were told to recall the two behaviors the social target performed (e.g., one for each separate prediction and outcome trial associated with each target). The recall phase was self‐paced.

## 3 RESULTS

In this section, we report the results from the prediction and outcome phases, followed by the memory results for the recognition and recall tests. To ensure that participants had learned the core trait of the social targets, we first examined the proportion of trials in which participants selected the consistent behavior in the prediction and outcome phase of the experiment (e.g., the proportion of time participants predicted the behavior that aligned with the core trait associated with targets in the person learning phase). Participants selected the consistent response 83.3% of the time, suggesting they had learned the core trait associated with each social target and were using that trait information in service of predictions.^3^

Turning to the memory phase of the experiment, we first report data from the recognition test by comparing the proportion of correctly recognized trials for trait‐consistent relative to trait‐inconsistent outcome trials. Trials were scored as correct if participants correctly identified the actual behavior (e.g., prediction outcome) the social target performed. Paired‐samples *t*‐test revealed that trait‐consistent outcomes (*M* = 0.89, *SD* = 0.13) were better remembered than trait‐inconsistent outcomes (*M* = 0.69, *SD* = 0.27), *t*(22) = 3.55, *p* = .002, *d* = .739.^4^, ^5^, ^6^

Turning to recall, we assessed participants’ ability to remember the behaviors the social targets performed in the prediction and outcome phases of the experiment (e.g., the two behaviors social targets actually performed). To do so, we took the recall responses and analyzed them according to the pre‐determined idea units (Bransfords & Franks, 1971). Specifically, an idea unit was scored as correct if the wording exactly matched the prediction outcome (i.e., the behavior the target actually performed). For example, if an outcome contained the idea unit “new neighbor,” then participants had to produce this exact phrase to receive a correct response for that unit. Misspelled words were counted as correct. We then analyzed the proportion of idea units correctly recalled for trait‐consistent and trait‐inconsistent outcome trials. The paired‐sample *t*‐test revealed no significant difference between the trait‐consistent (*M* = 0.284, *SD* = 0.197) and trait‐inconsistent outcomes (*M* = 0.260, *SD* = 0.20), *t*(22) = 0.94, *p* = .357, *d* = .196.

## 4 DISCUSSION

In this investigation, we examined episodic memory for outcomes that were consistent or inconsistent with predictions to better understand the relationship between future thinking and episodic memory. Specifically, we examined the extent the outcome of predictions, whether consistent or inconsistent with what participants predicted, affected how outcomes were subsequently remembered after the predicted events took place. We have two primary findings. First, we found that trait‐consistent outcomes were better remembered than trait‐inconsistent outcomes as measured by recognition, suggesting that information that is in line with what one knows is well‐remembered. This finding fits with past social cognitive work showing that schematic representations of targets affect memory (Judd & Kulik, 1980; Rothbart et al., 1979; Taylor & Crocker, 1981; Wyer & Srull, 1989), leading to enhanced memory for information that is consistent with what one knows about targets. Second, for recall, we found no difference in memory for trait‐consistent versus trait‐inconsistent outcomes. Overall, these data advance understanding about future thinking and how future thinking has an effect on episodic memory once predicted events play out.

The primary finding of this investigation is that memory for trait‐consistent outcomes was better than memory for trait‐inconsistent outcomes as measured by recognition. Such a finding is in line with past work showing that information about social targets consistent with what one already knows sometimes shows an advantage in memory. Theoretical accounts of person memory (e.g., how information about social targets is represented in episodic memory) suggest that people develop schematic representations about social targets based on prior experiences. These schematic representations can contain a range of different types of information about targets from specific behaviors targets have performed (“This person went over to greet the new neighbor that had moved in next door”) to less detailed information such traits targets exhibit across situations (“This person is extroverted”; Wyer & Srull, 1989). Once formed, such schematic representations are then useful in processing new information about targets. Importantly, these schema accounts posit that new information consistent with existing schema tends to be more easily remembered because that information can be well‐integrated into existing memory stores. Turning back to the present study, participants in our investigation developed a schematic representation of targets in the person learning phase (based on behaviors targets performed that implied a single trait, e.g., highly agreeable), used those representations to make predictions, and then integrated the outcomes of the predictions into episodic memory. Thus, our finding of better memory for trait‐consistent outcomes is in line with schema accounts. Finding better memory for trait‐consistent outcomes is further in line with the results of Frankenstein et al. (2020), who found better memory for outcomes consistent with predictions. Further, because the stimuli we used contained additional trait information associated with targets, the current results extend the findings of Frankenstein et al. (2020), which suggests that the advantage of trait‐consistent outcomes is generalizable across a range of different types of information associated with targets. Overall, our recognition results of a trait‐consistent memory advantage in episodic memory extend and replicate past work on future thinking by showing that the outcome of predictions influences how that information is subsequently stored in episodic memory.

Although we found evidence that trait‐consistent outcomes were better remembered than trait‐inconsistent outcomes, these results stand in contrast to abundant social cognitive work suggesting that information that is inconsistent with what one knows about targets is typically better remembered than information that is consistent (Hastie & Kumar, 1979; Rojahn & Pettigrew, 1992; Srull, 1981; Stangor & McMillan, 1992). Perhaps some of the strongest evidence that inconsistent information has a strong effect on memory (in tasks that do not involve making predictions) comes from meta‐analytic reports. Two different comprehensive meta‐analyses (Rojahn & Pettigrew, 1992; Stangor & McMillan, 1992) both support the idea that inconsistent information is generally better remembered than consistent information in the social domain. Interestingly, Rojahn and Pettigrew (1992) examined experimental factors that influenced the size of inconsistent memory effects in memory (e.g., how large the effect size is for inconsistent relative to consistent information) and found that the magnitude of such effects depended on different factors. Three of the factors they found to affect the size of the inconsistent advantage in memory were *number of dimensions* associated with targets (i.e., whether targets were associated with one core characteristic, two characteristics, or three or more characteristics), *proportion of irrelevant information* associated with targets, and *proportion of inconsistent to consistent information* associated with targets. For the first two factors (number of dimensions; proportion of irrelevant information), Rojahn and Pettigrew found that inconsistent memory effects were largest in experiments where there was only one core trait associated with targets and where there was no irrelevant information associated with targets as part of the experimental procedures. Importantly, the procedures we used in the current investigation where targets were only associated with one core characteristic (e.g., high agreeableness, etc.) and no irrelevant information may mean that we designed an experiment optimized to find a memory advantage for trait‐inconsistent information if such an advantage exists. For the third factor, the proportion of inconsistent to consistent information, Rojahn and Pettigrew found that inconsistent memory effects were largest when inconsistent information was relatively infrequent. Given that we used a procedure with equal numbers of trait‐consistent versus trait‐inconsistent outcomes, this may have limited our ability to find effects of prediction‐inconsistent outcomes on episodic memory, if such an effect exists. It is worth noting, however, that using a procedure where trait‐inconsistent outcomes are infrequent may be challenging to interpret because it is possible that improved memory for infrequent, inconsistent outcomes may be due to such information “sticking out” and being more distinctive in memory purely because it is rare (Fabiani & Donchin, 1995) and not because the information is inconsistent with memory stores requiring further processing (leading to enhanced memory). Future work may be necessary to understand the conditions under which a memory advantage for trait‐inconsistent outcomes may be more memorable than trait‐consistent information. For instance, one potential way to enhance memory for trait‐inconsistent outcomes is to manipulate how important, or salient to the self, the prediction task is. Neurobiological evidence suggests that making errors in predictions (i.e., prediction errors, which would be similar to the trait‐inconsistent outcomes) have the strongest effect on cognition when the task is highly salient to the self (Joiner et al., 2017; Schultz & Dickinson, 2000; Sel et al., 2016). This is relevant because in the current investigation, participants were making predictions about targets that did not involve the self, which may have made the prediction task lower in self salience. Thus, future work might deploy procedures that enhance the self‐salience of the prediction task, such as those used in investigating memory for self‐relevant information (i.e., self‐reference effects; Brown et al., 1986; Burden et al., 2021; Gutchess et al., 2007; Ilenikhena et al., 2021; Jackson et al., 2018; Leshikar & Duarte, 2012, 2014; Leshikar & Gutchess, 2015; Leshikar et al., 2015; Leshikar et al., 2016, 2017; Rogers et al., 1977; Symons & Johnson, 1997; Wong et al., 2017).

In this investigation, we observed a recognition memory advantage for trait‐consistent outcomes relative to trait‐inconsistent outcomes, offering an advance in understanding how episodic memory and future thinking are related. Recent theoretical work in future thinking has made progress in identifying different varieties or “taxonomies” of future thinking (Szpunar et al., 2014). Specifically, this work posits that future thoughts can be of the following types: simulations (creating a mental image of a future event), predictions (judging what is likely to happen in a future event), intentions (making a plan to accomplish something in the future), or planning (generating specific steps to accomplish something in the future). This framework also suggests that these different varieties of future thinking can draw upon either episodic memory representations (i.e., specific details of past experiences) or semantic memory representations (i.e., gist‐like representations abstracted from past experiences), or both. Turning back to the current investigation, our findings of a memory advantage for trait‐consistent outcomes can be interpreted through this taxonomy. Specifically, participants in this investigation were making *predictions* (judging what is likely to happen in the future) based on *episodic memory* representations (acquired in the person learning phase of the experiment). Thus, our results extend knowledge on how *episodic* memory is used in making *predictions*, as a variety of future thinking, as well as how the outcome of those predictions is then subsequently stored in *episodic* memory after the predicted event plays out. Future work should investigate the influence of prediction outcomes on memory for other varieties of future thinking such as simulations, and they should further do so in tasks that are more reliant on semantic memory representations to better understand the relationship between future thinking and memory. Understanding episodic memory is an important pursuit (Jennings & Jacoby, 1993; Giannakopoulos et al., 2021; Leach et al., 2018; Matzen et al., 2015; McCurdy, Frankenstein, et al., 2020; McCurdy & Leshikar, in press; McCurdy, Viechtbauer, et al., 2020; McCurdy et al., 2019, 2017, 2021; Yonelinas, 2002), and this work contributes to that effort.

Turning to our recall findings, we found no evidence that prediction outcome affected subsequent episodic memory. Past work on memory for inconsistent versus consistent information associated with targets (in procedures that do not involve predictions) sometimes shows different patterns of results depending on whether memory is measured via recognition or recall as demonstrated by meta‐analytic evidence (Rojahn & Pettigrew, 1992; Stangor & McMillan, 1992). For instance, in one investigation, participants were shown targets who were either intelligent or hostile, along with additional behavioral and trait information that was either consistent or inconsistent with that core characteristic (e.g., intelligent, hostile). Then, memory for these details was measured by both recognition and recall procedures. The results showed that memory was better for consistent, compared to inconsistent information as measured by recognition, whereas memory was better for inconsistent, compared to consistent information as measured by recall (Wyer et al., 1984). Such differences in memory performance may be due to differences in retrieval processing demands for recognition versus recall measures (Gillund & Shiffrin, 1984). Although we expected to see similar effects for memory as measured by both recognition and recall, we found no memory effects as measured by recall. It may be that our recall test was not sufficiently sensitive to measure differences between trait‐consistent versus trait‐inconsistent outcomes. Future work may be necessary to test this possibility.

In this investigation, we found evidence that memory was better for trait‐consistent relative to trait‐inconsistent outcomes. Interestingly, the two prediction outcome types we used in this experiment (trait‐consistent, trait‐inconsistent) may be related to principles in prediction error and reward learning literatures. Work on prediction errors and reward learning suggests that there are different varieties or types of prediction errors. One such distinction is that there are both positive and negative prediction errors (Rouhani & Niv, 2021). Positive prediction errors arise when organisms make a prediction and have some uncertainty regarding whether their prediction is correct before learning that their predictions are indeed accurate (i.e., their prediction is better than expected). Negative prediction errors arise when organisms make a prediction and that prediction is later inaccurate (i.e., their prediction is worse than expected). Interestingly, improved memory for positive prediction error events may be associated with increased dopamine release, which then may modulate activity in memory‐related brain regions, such as the hippocampus (Rouhani & Niv, 2021; Rouhani et al., 2018; Schultz & Dickinson, 2000). Turning back to the present study, it is possible, therefore, that our trait‐consistent outcomes may be thought of as positive prediction errors, and further, our trait‐inconsistent outcomes may be considered negative prediction errors. Although speculative, if our trait‐consistent outcomes are indeed positive prediction errors, then it is possible that the improved memory for target‐consistent outcomes (e.g., positive prediction errors) we observed in this investigation may be due to the influence of dopaminergic activity on memory‐related brain regions such as the hippocampus. Future work might be necessary to investigate this possibility.

Past work has made the case that future thinking may serve an adaptive function: Thinking about what may happen in the future may lead to improved goal‐directed behaviors (Schacter et al., 2017). Thus, our finding of improved recognition memory for trait‐consistent outcomes (but not trait‐inconsistent), may reflect an adaptive function of memory. Over the course of a lifetime of experiences, people develop a well‐structured understanding of the world represented in memory that is often a good guide on what to expect in the future. Because people come to rely on such schematic representations, it may be that individuals strongly process outcomes in relation to information stored in memory (e.g., schemas), and because of this show substantially better memory for information consistent with schematic representations. Although speculative, it may take an especially surprising or unexpected event before people adjust their schematic representation to incorporate inconsistent information into memory. Barring an unusually surprising outcome, however, people may rely on existing schemas to process incoming information to understand their environment. Overall, this investigation adds to work in other domains suggesting that memory serves an adaptive function (Bell & Buchner, 2012; Kadwe et al., 2022; Meyer et al., 2020; Nairne et al., 2007; Sklenar et al., 2021; Udeogu et al., 2022; Villasenor et al., 2021), which may allow people to successfully navigate their social world.

Although we found evidence that trait‐consistent outcomes were better remembered than trait‐inconsistent outcomes, at least as measured by recognition, there are two limitations of the investigation worth describing. First, as mentioned above, our trait‐consistent and trait‐inconsistent outcomes could be thought of as positive and negative prediction errors, respectively. Because of the way we experimentally manipulated prediction outcomes (e.g., whether behaviors were consistent or inconsistent with the core trait associated with targets), this means that the type of prediction error (positive or negative) was confounded with consistency (i.e., trait‐consistent outcomes were always a positive prediction error, and trait‐inconsistent outcomes were always negative prediction errors). Turning back to the results of this experiment, what this may mean is that improved memory for trait‐consistent outcomes could be due to consistency effects (e.g., better memory for consistent information) or it could be due to positive prediction error effects (e.g., improved memory for positive prediction errors). Future work may be necessary to dissociate consistency from positive prediction effects on memory for prediction outcomes to better understand the relationship between prediction outcomes and memory. Second, in our procedures, prediction outcomes were based on the core trait associated with the target (and not based on participants’ responses in the prediction and outcome phase of the experiment, per se). What this means is that not all participants saw half prediction‐consistent and half prediction‐inconsistent outcomes for each target (in the prediction and outcome phase of the experiment), which could have affected our memory results. We see this as less likely, however, because in an additional analysis, we examined memory effects only for the subset of trials where participants correctly chose the behavior consistent with the core trait associated with targets, and the results of that additional analysis were in line with our primary analyses.

In conclusion, we found evidence that the outcome of predictions affects how those events are remembered subsequently. Specifically, we found that trait‐consistent outcomes were better remembered than trait‐inconsistent outcomes as measured by recognition, which may reflect an adaptive use of memory. Although past work suggests that the contents of episodic memory are used in future thinking, the results of the current study add to past work by demonstrating that the outcome of predictions has an influence on episodic memory, which advances our understanding of the relationship between episodic memory and future thinking.

## CONFLICT OF INTEREST

The authors have no conflicts of interest to report.

## Supporting information



Supporting information.
**APPENDIX** Depiction of information associated with targets in the person learning phase of the experiment.

## Data Availability

Data will be made available upon reasonable request.

## ENDNOTES

1 We piloted these procedures in an independent sample of participants. The results of the pilot for our comparison of interest (memory for trait‐consistent vs. trait‐inconsistent outcomes) show an effect size of *d* = .92, but we used a more conservative effect size (*d* = .6) to perform this power analysis.

2 Prediction outcomes were pre‐programmed based on the core trait associated with each target, regardless of which behavior the participant selected in the prediction task.

3 As part of the prediction and outcome phase of the experiment, participants made expectancy judgments where they reported whether they “expected” the outcome the target actually performed (e.g., prediction outcome), after they learned which behavior the target actually performed. Participants selected “yes” (i.e., expected) 83.1% of the time for the trait‐consistent outcomes, and further they selected “no” (i.e., unexpected) 78.6% of the time for trait‐inconsistent outcomes. These results are further in line with the idea that participants had learned the core trait for each social target and used that memory representation to make predictions about targets.

4 Because participants made predictions consistent with the core trait for each participant 83.3% of the time, this means that for a small percent of trials, participants predicted behaviors that were not in line with the core trait for that target. To account for this, we reanalyzed the recognition data using only those trials where participants correctly predicted the outcomes consistent with the core trait of the target. Paired‐samples *t*‐test again revealed the same pattern where trait‐consistent outcomes were better remembered than trait‐inconsistent outcomes, *t*(22) = 4.56, *p* < .001, *d* = .951.

5 Given that participants made expectancy judgments after they found out the behavior each target performed (i.e., prediction outcome), it is also possible to analyze memory performance in terms of consistency (trait‐consistent, trait‐inconsistent) as well as expectancy (expected, unexpected). In Frankenstein et al., 2020, they reported a Consistency by Expectancy interaction where memory was enhanced for inconsistent trials that were also subjectively rated as unexpected. Although this was not the focus of the present study, we performed an analogous analysis. The results indicated that the consistency by expectancy interaction was significant, *F*(1,22) = 53.36, *p* < .001, which was driven by improved memory for trait‐inconsistent outcomes when those trials where subjectively endorsed as unexpected.

6 As part of our procedures, each social target was associated with one consistent and one inconsistent prediction outcome. This means that half the targets were first associated with a trait‐consistent outcome, and the remaining half of the targets were first associated with a trait‐inconsistent outcome. Because of this, it is possible that different memory effects might be observable depending on whether targets were first associated with a trait‐consistent or trait‐inconsistent outcome. To examine this possibility, we performed a 2 (Consistency: Consistent or Inconsistent) x 2 (Presentation: Consistent First or Inconsistent First) repeated‐measures analysis of variance on our recognition data. The results showed a main effect for consistency, *F*(1,22) = 12.58, *p* = .002, with trait‐consistent outcomes better remembered than trait‐inconsistent outcomes, in‐line with our primary analyses. There was no main effect for presentation, *F*(1,22) = 0.43, *p* = .520, and no significant interaction between consistency and presentation, *F*(1,22) = 0.49, *p* = .490.
