# Supplementary figures and images for: The reciprocal relationship between episodic memory and future thinking: How the outcome of predictions is subsequently remembered
Source: Brain Behav. 2022 Aug 24;12(9):e2603. doi: 10.1002/brb3.2603 (PMC9480898; doi:10.1002/brb3.2603)

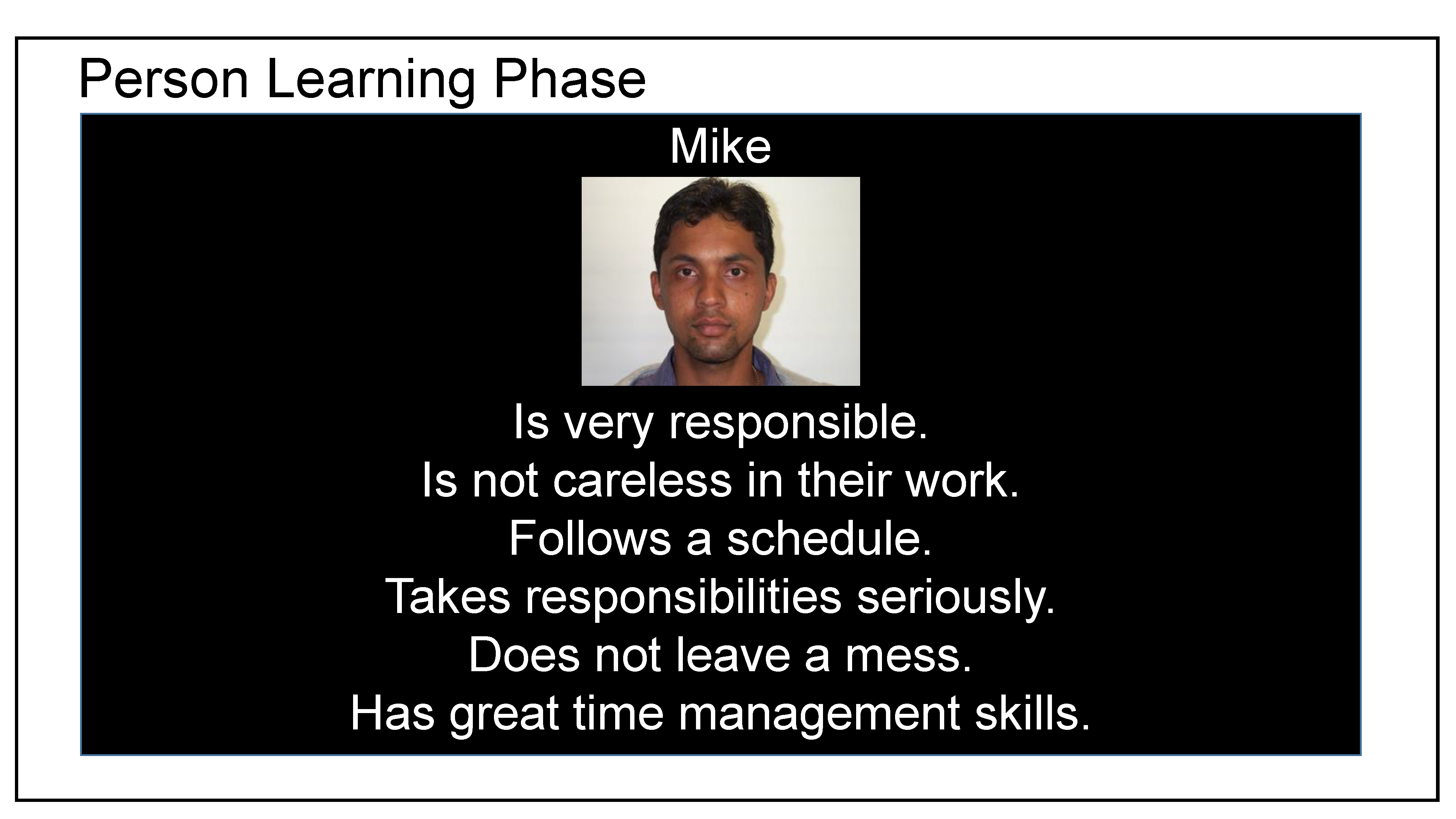

Supplement: Supplementary file 1 — Supporting information. APPENDIX Depiction of information associated with targets in the person learning phase of the experiment. [file BRB3-12-e2603-s001.tif]
